# Editorial: Prescription digital therapeutics in psychiatry

**DOI:** 10.3389/fpsyt.2024.1550666

**Published:** 2025-01-10

**Authors:** Brett M. Colbert, Derek M. Dykxhoorn, Shaheen Lakhan

**Affiliations:** ^1^ Click Therapeutics, Inc., New York, NY, United States; ^2^ Leonard M. Miller School of Medicine, University of Miami, Miami, FL, United States

**Keywords:** digital therapeutic, virtual reality, regulatory guidance, cognitive behavioral therapy (CBT), PDURS

The integration of Prescription Digital Therapeutics (PDTs) into modern psychiatric care marks a transformative moment in healthcare innovation. PDTs are software-based treatments designed to address a wide range of conditions, either as standalone therapies or in combination with traditional pharmacological treatments. Regulated by authorities such as the FDA as ‘Software as a Medical Device’ (SaMD), PDTs are reshaping psychiatric care by providing accessible, scalable, and evidence-based treatments for mental health disorders, including depression, anxiety, insomnia, and substance use disorders. This Research Topic in *Frontiers in Psychiatry* brings together a collection of articles that showcase the state-of-the-art advancements in PDT development, regulation, and real-world implementation.

With the growing burden of mental health disorders worldwide and persistent disparities in access to care, PDTs offer the potential to democratize treatment delivery. By leveraging information technology, PDTs address key challenges in health care delivery, including clinician shortages, treatment adherence, and personalization of care. In this editorial, we summarize the scientific contributions presented in this Research Topic and connect their findings to the broader landscape of digital therapeutics while highlighting the groundbreaking implications of the FDA’s recent *Prescription Drug Use-Related Software* (PDURS) guidance. This new regulatory milestone signals that the next big step in PDT adoption and integration has arrived.

## Summay of topic contributions

The five articles included in this Research Topic explore key aspects of PDTs, from clinical efficacy and engagement to regulatory frameworks and implementation science, offering a comprehensive overview of the current digital therapeutic landscape.

Thorndike et al. showcase the real-world efficacy of Somryst^®^, a PDT delivering digital Cognitive Behavioral Therapy for Insomnia (CBT-I). Results from the DREAM study revealed significant reductions in insomnia severity, depression, and anxiety symptoms over a one-year follow-up, highlighting the scalability and durability of PDTs in addressing sleep disorders, particularly for individuals with limited access to in-person care. Blanc et al. explore the promise of virtual reality-based PDTs through NurtureVR™, emphasizing its capacity to reduce stress among perinatal Black and Latina women. Their qualitative study identifies barriers such as accessibility and cultural relevance, advocating for equity-focused innovations to address mental health disparities in underserved populations. Similarly, Carrasco et al. present a protocol for evaluating NurtureVR™ in expectant and postpartum Black and Latina mothers, integrating mindfulness, relaxation, and guided imagery techniques to tackle significant disparities in maternal mental health outcomes. This study emphasizes the importance of culturally tailored digital interventions in perinatal care.

Expanding on the foundational elements of PDTs, Ferrante et al. discuss the NIH’s role in supporting PDT development for conditions like depression, substance use, and cognitive disorders. They highlight regulatory hurdles and the necessity for sustained research funding to ensure widespread adoption and equitable implementation. Docherty et al. provide a historical perspective, tracing the evolution of psychotherapy from Freudian analysis to the emergence of PDTs. By situating PDTs within this broader context, they argue that digital therapeutics represent a natural progression of evidence-based care, bridging gaps in accessibility while retaining fidelity to therapeutic principles. Together, these articles illuminate the dynamic intersections of clinical efficacy, equity, innovation, and historical evolution, showcasing how PDTs are reshaping mental health care and laying a foundation for future advancements.

## The PDURS guidance: a regulatory milestone

The articles in this Research Topic provide important insights into the development, implementation, and outcomes of PDTs. It is clear that the field has now entered a pivotal phase in its development. The recent release of the FDA’s *Prescription Drug Use-Related Software* (PDURS) guidance is a watershed moment for PDTs. This guidance provides a clear regulatory pathway for software and prescription drug combinations—whether the software enhances drug delivery and efficacy, monitors adherence, or supports clinical decision-making.

The PDURS guidance directly addresses two critical challenges that have limited PDT integration into routine care – regulatory ambiguity and clinical validation. First, it clarifies how end-user outputs from PDTs are categorized as either FDA-required labeling or promotional labeling. For instance, if software functions provide a clinically meaningful benefit, such outputs can be included in the prescribing information (PI) and are subject to FDA review as part of the drug’s approval process. This offers an unprecedented opportunity for PDT developers to align their software with the same evidentiary standards as pharmacological treatments. This guidance opens the door for software-enhanced drugs tailored to both new drugs in development, as well as older drugs that are facing adoption or patent life-cycle challenges.

Second, the PDURS guidance underscores the importance of demonstrating clinically meaningful outcomes. For PDTs to be included in FDA-required labeling, sponsors must conduct rigorous, well-controlled studies showing that the software provides measurable improvements in patient outcomes. This creates a framework for high-quality evidence generation, ensuring that PDTs meet the same safety and efficacy standards as traditional therapeutics.

The implications of these new guidelines for psychiatry are profound. PDTs are uniquely suited to address mental health disorders, where outcomes often depend on complex interactions between behavioral, neurobiological, and environmental factors. With the PDURS framework in place, PDTs can now be developed, validated, and prescribed alongside pharmacological treatments, enabling a truly integrated approach to psychiatric care. This will not only enhance patient outcomes but also provide clinicians with new tools to deliver precision medicine at scale.

## Looking ahead: the future of PDTs in psychiatry

The articles in this Research Topic, including the historical perspective provided by Docherty et al., combined with the regulatory clarity offered by the PDURS guidance, signal that PDTs have reached an inflection point. By grounding the evolution of PDTs within a historical trajectory, Docherty et al. remind us that the field builds on a strong foundation of evidence-based care, even as it moves toward innovative digital solutions for delivery of this care. Moving forward, several key priorities will shape the trajectory of PDTs in psychiatry:

1. Integration into Clinical Practice: With regulatory pathways clarified, the next challenge lies in integrating PDTs into routine psychiatric practice. As highlighted by Ferrante et al., collaboration between developers, clinicians, and policymakers will be essential to navigate implementation barriers, including clinician education and reimbursement strategies. The real-world success of Thorndike et al.‘s DREAM study demonstrates the potential for PDTs to achieve sustained clinical impact in real-world settings, providing an effective template for integration.
2. Equity and Accessibility: As highlighted by Blanc et al. and Carrasco et al., PDTs must be designed with inclusivity in mind. Future efforts should focus on addressing barriers to access, such as digital literacy, cultural relevance, and affordability, especially for minoritized communities. The promising applications of VR-based PDTs for perinatal mental health underscore the importance of culturally tailored solutions.
3. Equity and Accessibility: Robust clinical trials and real-world evidence will be critical to building trust in PDTs. The DREAM study by Thorndike et al. sets a strong precedent for how PDT efficacy can be rigorously evaluated and demonstrated. Ferrante et al. further emphasizes the need for sustained funding and infrastructure to support PDT research across diverse populations and conditions.
4. Technological Innovation: Emerging technologies such as artificial intelligence, virtual reality, and wearable sensors hold great promise for advancing PDTs. The work by Carrasco et al. and Blanc et al. demonstrates how VR can be leveraged to address stress and improve outcomes in perinatal care. These innovations will enable even greater personalization, monitoring, and optimization of care, advancing the overall effectiveness of PDTs.

## Conclusion

Prescription Digital Therapeutics represent a transformative innovation in psychiatric care, offering scalable, evidence-based solutions to some of the most pressing challenges in mental health. The articles in this Research Topic illustrate the remarkable progress being made in PDT development, regulation, and implementation. Yet it is the FDA’s PDURS guidance that heralds the next big step in the integration of PDTs into clinical medicine. PDTs are poised to be integrated into clinical practice alongside pharmacological treatments, validated by rigorous evidence, and recognized as essential components of patient care.

As we stand at this turning point, it is clear that the future of PDTs in psychiatry is brighter than ever. By embracing this new paradigm, clinicians, researchers, and regulators can work together to ensure that PDTs fulfill their potential to improve mental health outcomes for all.

